# Clinical features and outcome of adult T-cell leukemia/lymphoma: University of Miami experience

**DOI:** 10.1186/1742-4690-11-S1-O1

**Published:** 2014-01-07

**Authors:** Agustin Pimentel, Isildhina Reis, Jennifer R Chapman-Fredricks, Juan Carlos Ramos

**Affiliations:** 1 Division of Hematology-Oncology, Department of Medicine, Sylvester Comprehensive Cancer Center, University of Miami, Miami, FL, USA; 2 Department of Epidemiology & Public Health, University of Miami Miller School of Medicine and Jackson Memorial Hospital, Miami, FL, USA; 3 Department of Pathology and Laboratory Medicine, University of Miami Miller School of Medicine and Jackson Memorial Hospital, Miami, FL, USA

## 

Adult T-cell leukemia/lymphoma (ATLL) is an aggressive malignancy with a poor prognosis caused by HTLV-I. Miami is proximal to the Caribbean, where HTLV-I is endemic. We have identified at least 140 cases between 1987 and 2013. A total of 108 patients have been analyzed for treatment response, including 51 acute, 50 lymphomatous, 5 chronic (4 unfavorable), and 2 smoldering types. The median overall survival for acute and lymphomatous was 6 and 10 months respectively, and not reached for chronic and smouldering types. Fifty-one patients (33 acute, 11 lymphomatous, 5 chronic, and 2 smouldering) were treated with high-dose AZT/interferon (IFN) as first line therapy. The complete and overall response rates (CR and ORR) for acute/unfavorable chronic vs. lymphomatous types were 27% vs. 9%, and 57% vs. 9% respectively. Three out of 4 (75%) unfavorable chronic type patients responded to AZT/IFN (2 CR, and 1 partial response). Seventy-three patients received chemotherapy at some point during their treatment. The CR rate and ORR for acute/unfavorable chronic vs. lymphomatous-type patients treated with chemotherapy-based regimens were 33 % vs. 17%, and 60% vs. 73% respectively. Five acute type patients who had failed AZT/IFN achieved remission after chemotherapy. By contrast, patients treated with AZT/IFN as second line therapy generally did not respond. Finally, we observed several long-sustained responses in acute and unfavorable chronic subtypes treated with first line AZT/IFN alone translating into a survival benefit. A comprehensive stratified analysis of clinical characteristics, pertinent immunophenotypic markers, and treatment outcome will be presented at the meeting.

